# The HEALERS: a patient, community, and stakeholder advisory board focus group series to refine a novel virtual world-based cardiac rehabilitation intervention and clinical trial

**DOI:** 10.3389/fdgth.2025.1427539

**Published:** 2025-07-15

**Authors:** Helayna Abraham, Grace Patrice Anyetei-Anum, Ashton Krogman, Donald Clark, Melvin Echols, Michael E. Hall, Karen Hodgman, Brian Kaihoi, Stephen Kopecky, Shawn Leth, Shaista Malik, Jill Marsteller, Lena Mathews, Robert Scales, Phillip Schulte, Adam Shultz, Julie Becker, Bryan Taylor, Randal Thomas, Nathan D. Wong, Thomas Olson, LaPrincess C. Brewer

**Affiliations:** ^1^ Department of Internal Medicine, Division of Cardiology, Baylor Heart and Vascular Institute, Dallas, TX, United States; ^2^ Mayo Clinic Alix School of Medicine, Mayo Clinic, Rochester, MN, United States; ^3^ Department of Cardiovascular Medicine, Mayo Clinic College of Medicine, Rochester, MN, United States; ^4^ Division of Cardiology, University of Mississippi Medical Center, Jackson, MS, United States; ^5^ Department of Cardiovascular Medicine, Morehouse School of Medicine, Atlanta, GA, United States; ^6^ Global Products and Services, Mayo Clinic Center for Innovation, Rochester, MN, United States; ^7^ Division of Cardiology, Department of Medicine, University of California, Irvine, CA, United States; ^8^ Center for Health Services and Outcomes Research, Johns Hopkins Bloomberg School of Public Health, Baltimore, MD, United States; ^9^ Division of Cardiology, Johns Hopkins School of Medicine, Baltimore, MD, United States; ^10^ Department of Cardiovascular Medicine, Mayo Clinic College of Medicine, Phoenix, AZ, United States; ^11^ Division of Clinical Trials and Biostatistics, Mayo Clinic, Rochester, MN, United States; ^12^ Department of Cardiovascular Medicine, Mayo Clinic College of Medicine, Jacksonville, FL, United States; ^13^ Center for Clinical and Translational Science, Mayo Clinic, Rochester, MN, United States

**Keywords:** cardiac rehabilitation, telehealth, virtual world, community engaged research, healthcare equity patient, caregiver, stakeholder advisory board

## Abstract

**Background:**

Cardiac rehabilitation (CR) is a widely underutilized secondary cardiovascular disease prevention strategy, due to a variety of barriers to participation that disproportionately impact women, minoritized racial and ethnic groups, and patients with low socioeconomic status. *Destination Cardiac Rehab*, a virtual world-based CR (VWCR) program designed by our team in collaboration with patients and community members to mitigate the barriers to CR participation, has demonstrated feasibility and acceptability. In anticipation of a randomized controlled trial (RCT) to further validate the intervention, this qualitative descriptive analysis provides insights garnered from a patient/community/stakeholder-advisory board (PCS-AB, HEALERS) focus group series, convened to inform iterative refinements to a RCT protocol.

**Methods and results:**

HEALERS participated in five 90-min virtual focus group sessions to provide feedback on various aspects of the VWCR intervention and the recruitment/retention strategies. Major themes were identified from participant feedback to inform revisions to the trial protocol. Illustrative quotes were selected to represent each theme. Twenty-two members were recruited with diverse sociodemographic and personal/professional backgrounds (mean age 59.3 ± 13 years, 50% female). Regarding trial recruitment, members recommended effective communication strategies, recruitment video suggestions, and expansion of recruitment settings. HEALERS emphasized the importance of feeling safe during exercise and social support in designing an effective VWCR intervention. Lastly, they identified reminder messages, tangible incentives, and fostering positive relationships with the CR staff as important retention tools.

**Conclusions:**

A diverse PCS-AB was convened to better understand community needs to improve the patient-centric nature of *Destination Cardiac Rehab* in anticipation of an upcoming RCT. The HEALERS offered valuable insights that informed actionable changes to the RCT protocol.

## Introduction

Community-engaged research (CER) approaches engage community members, patients and key stakeholders in collaboration with study teams to provide insight into community needs and promote healthcare equity (1). The literature demonstrates tremendous value in incorporating community members in the development and execution of novel digital health interventions in cultivating trusting relationships between patients and the health care team, to address health disparities, and ensure culturally sensitive interventions that better address patient needs (1–7). Patient advisory boards have been shown to optimize study design, increase diversity, and foster trust leading to more effective and impactful healthcare solutions (8–12). In collaboration with community members, our study team developed a virtual world-based cardiac rehabilitation (VWCR) program, *Destination Cardiac Rehab*, to address often overwhelming barriers to participation in traditional center-based cardiac rehabilitation (CBCR) of which <25% of eligible patients participate in despite extensive evidence demonstrating its benefits (13–19). These barriers are particularly burdensome for minoritized racial and ethnic groups, women, and patients with low socioeconomic status (SES) (20–22). Alternative platforms for cardiac rehabilitation (CR) delivery that provide more flexibility, such as home-based CR (HBCR) programs, have emerged in an effort to mitigate the barriers to CR participation but have not substantially improved CR participation (23–26). Incorporating mobile and internet technologies into these programs has shown promise in broadening CR engagement (27, 28).

**Figure 1:**
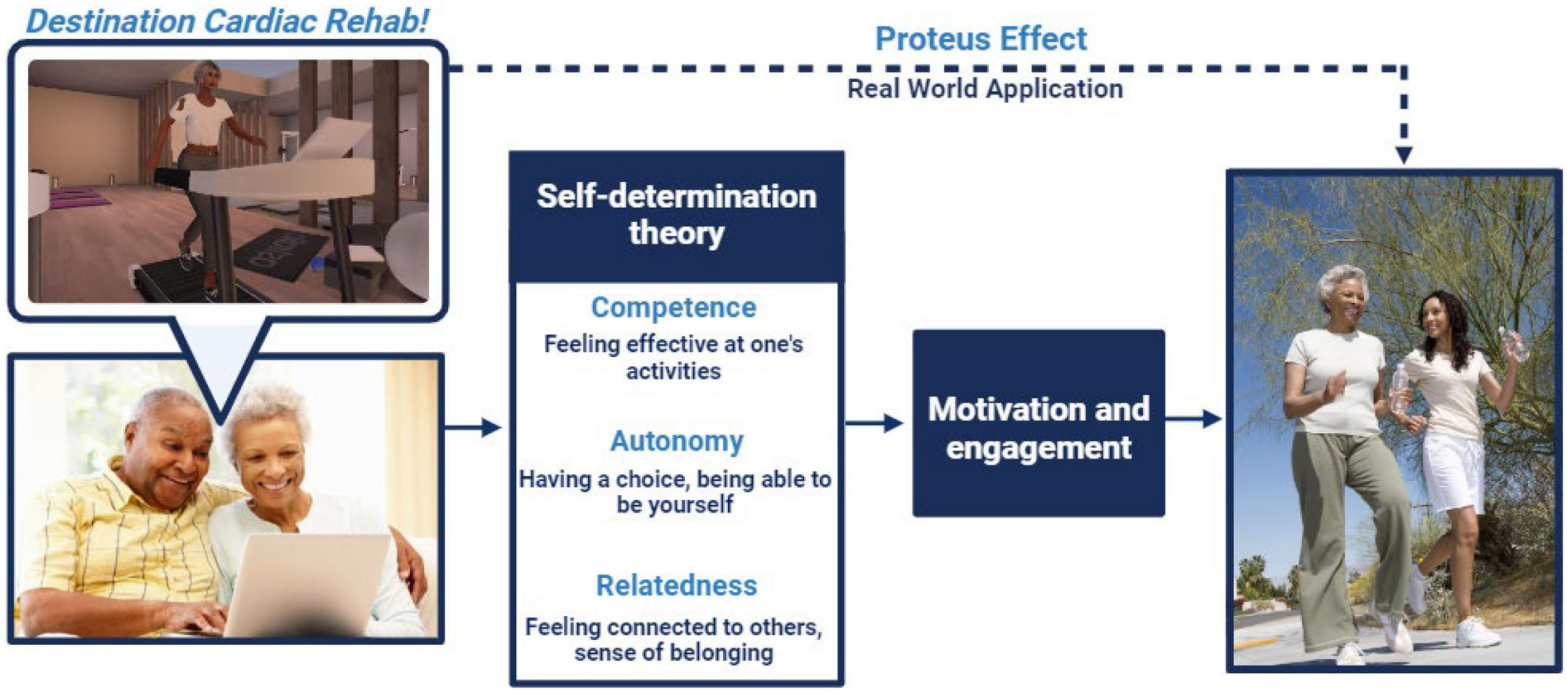
Theoretical framework for destination cardiac rehab. Created with BioRender.com. Bottom left image: Reproduced with permission from “Senior African American couple using laptop” by Monkey Business Images, licensed under Standard Image License. Right image: Reproduced with permission from “Motion blur shot of an African American mother and daughter jogging together in park” by sirtravelalot, licensed under Standard Image License.

*Destination Cardiac Rehab* takes place in an immersive VW platform, Second Life®, and utilizes virtual avatars to simulate a real-world experience but from the convenience of any location (29). VW interventions capitalize on a phenomenon known as the “Proteus effect”, in which individuals integrate the behaviors and characteristics of their avatars in the VW into their own behaviors and self-perception in the real world (Figure 1) (30). The intervention was designed to employ the tenets of self-determination theory which posits that competence, autonomy, and relatedness motivate behavioral change (31). Prior proof-of-concept and pilot studies have demonstrated acceptability, feasibility, excellent participation and adherence rates, and high user satisfaction of the VWCR intervention (32, 33). The pilot study additionally demonstrated a trend toward improvements in cardiovascular health (CVH) behaviors (33). Participant feedback from the two prior studies have been used to refine the platform to better meet patients' needs. To validate *Destination Cardiac Rehab* as a viable alternative to traditional CBCR, our study team plans to conduct a randomized controlled trial (RCT) comparing participation and adherence rates and clinical outcomes in patients participating in *Destination Cardiac Rehab* vs. traditional CBCR (34).

### Context

**Figure 2:**
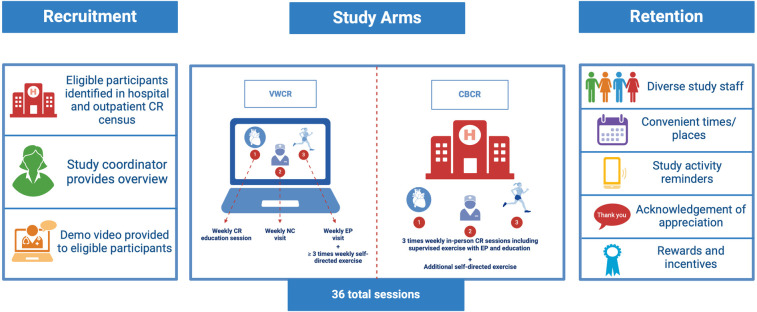
Overview of trial protocol including overview of intervention and control arms. Created with BioRender.com.

The upcoming RCT will recruit patients from six study sites (three Mayo Clinic sites: Rochester, MN, Phoenix, AZ, and Jacksonville, FL; Johns Hopkins Hospital, Baltimore, MD; University of California, Irvine, CA; and University of Mississippi Medical Center, Jackson, MS). Figure 2 provides an overview of the intervention, recruitment/retention strategies and a side-by-side comparison of CBCR vs. VWCR. Eligible participants will be identified by the study coordinators both in the inpatient setting from the hospital service census and outpatient CR enrollment lists. Once eligibility is confirmed, potential participants will be approached and provided with an overview of the study and demo video. Participants who consent to enrollment will be randomized to either the CBCR (control group) or *Destination Cardiac Rehab* (intervention group). A detailed description of the RCT protocol was previously published (34).

Briefly, participants in both groups will undergo an initial health assessment (e.g., clinical measures, laboratory studies, etc.) and form an individualized treatment plan (ITP) which will include relevant clinical history, exercise program description, risk factor modification plan, and psychosocial assessment. Participants randomized to the control group will participate in a standard, in-person, 36-session CBCR program over 12 weeks (3 sessions/week). Participants in the VWCR group will also engage in 3 virtual CR sessions per week over 12 weeks (total 36 sessions) including education sessions within the VW platform. The education session topics were previously published in the RCT protocol (34). Virtual visits with a nurse coach (NC) to review key concepts from the education sessions, CV symptoms, vital signs, and medications, and virtual visits with an exercise physiologist (EP) to review physical activity (PA) patterns and to receive a personalized exercise prescription. They may also attend an optional weekly peer-support group designed to mimic the social support experienced in an in-person environment. Participants in both groups will be encouraged to exercise on their own outside of the 3 standard exercise sessions per week.

Prior to commencement of the RCT, a patient/community/stakeholder advisory board (PCS-AB), self-dubbed the HEALERS (**H**eart **E**mpowerment and **A**dvisory for **L**ife **E**nhancement in Cardiac **R**ehab **S**ettings), was convened to further develop and refine the RCT recruitment strategy, *Destination* *Cardiac Rehab* intervention/platform, and retention strategy to better meet the needs of patients. As a part of the theoretical framework for CER, the Patient-Centered Outcomes Research Institute (PCORI) Engagement Rubric was utilized in the RCT planning phase. This rubric focuses on four principles: reciprocal relationships, partnerships, co-learning, and transparency to optimize meaningful patient and stakeholder involvement (35). The HEALERS will continue to meet quarterly throughout the 5-year trial to inform both the study conducting phase and disseminating the study results.

This qualitative descriptive analysis of a focus group (FG) series aims to: (1) describe the methods for recruiting the HEALERS, (2) outline the series structure, (3) and detail a pragmatic approach to the systemic analysis of feedback obtained during the series to inform iterative refinements and enhancements to *Destination Cardiac Rehab* and the forthcoming clinical trial recruitment and retention strategies (36).

## Methods

### Advisory board recruitment

The Mayo Clinic Institutional Review Board approved this study. The study team employed both purposeful and convenience sampling strategies to recruit patients from each of the six study sites who were previously eligible for or participated in CR programs in addition to caregivers and relevant administrators/stakeholders with expertise in CR administration to ensure a diverse group that represents the population of interest, in accordance with the PCORI Engagement Rubric. Eligibility criteria were the following: patients who were eligible for CR who completed CR, enrolled in but did not complete CR, and those who did not enroll in CR, caregivers of patients who were eligible for CR, and representatives from key stakeholder/advocacy groups (e.g., American Association of Cardiovascular and Pulmonary Rehabilitation, American Heart Association, Mayo Clinic Information Technology, and CR payers), age ≥18 years old, basic internet navigation skills, and an active email address.

Recruitment flyers with study team contact information and a detailed description of the advisory board purpose and activities were distributed to all six participating sites to recruit representation from diverse geographic locations. The flyers were displayed at the CR center of each site and distributed by CR staff to interested past and current patients. Patients, caregivers and stakeholders interested in participating voluntarily reached out to the study team contact and were screened for eligibility. Additionally, Mayo Clinic patients who had been referred to CR were screened for eligibility and approached by study team members with whom they had no prior relationship via phone, secure patient portal message, or email. Coercion was prevented as the recruitment materials were shared with anyone at the CR site, rather than approaching specific individuals. To further minimize coercion, any interested individuals were required to contact the study team on their own. Interested CR patients, caregivers and stakeholders contacted the study team by phone or email to confirm their interest and ability to complete all expected advisory board activities. Following eligibility screening conducted by the study coordinator via phone call, informed consent and HIPAA authorization forms were sent electronically. A Mayo Clinic approved process for documenting informed consent and HIPAA authorization was completed and enrolled participants were provided copies of the signed forms.

### Data collection

**Figure 3:**
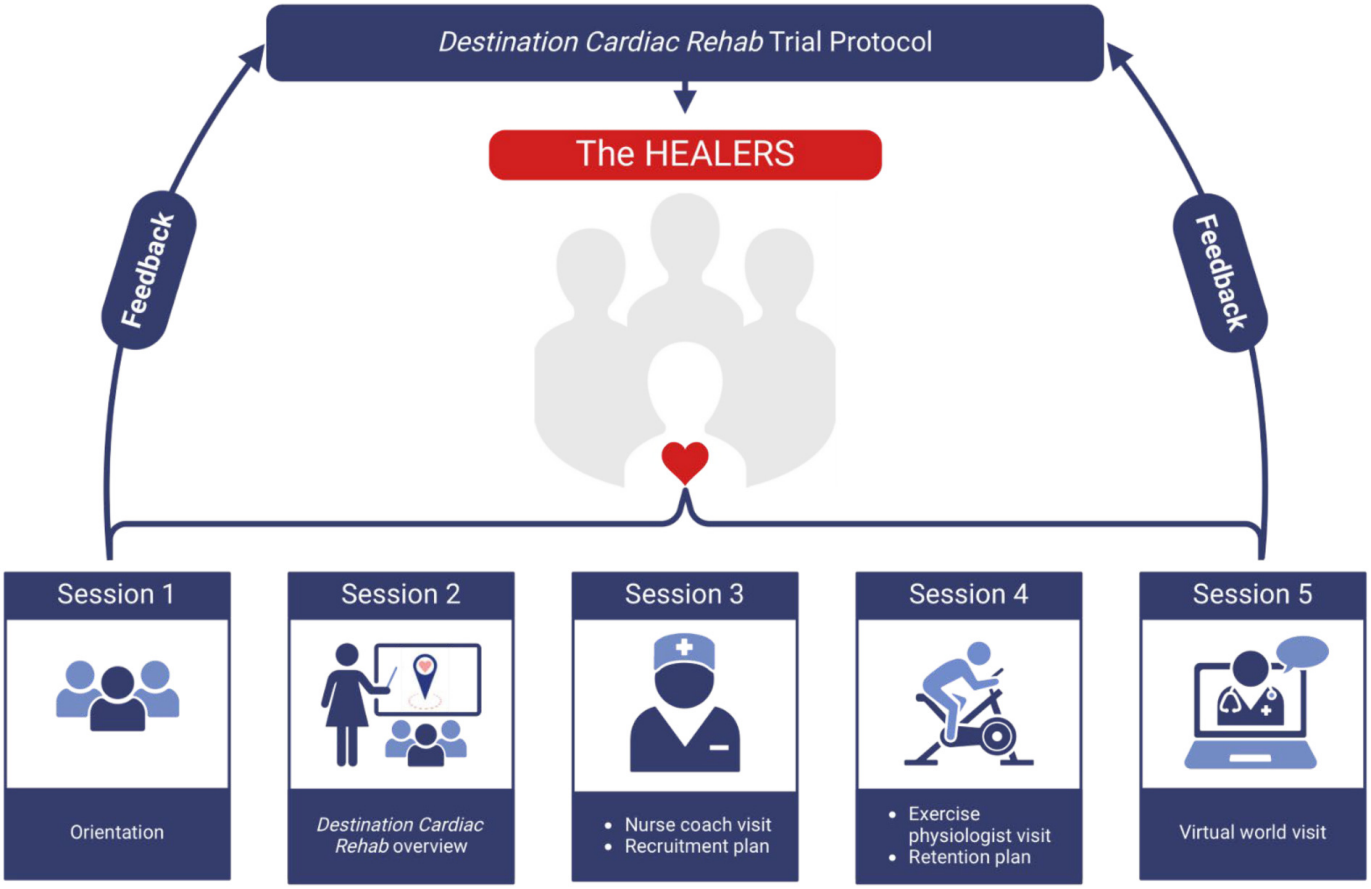
Study overview with a brief description of the FG session topics. Created with BioRender.com.

After advisory board recruitment was finalized, five 90-minute virtual FG sessions occurred monthly via videoconferencing (Zoom©). The FG moderator guides and agendas were informed by the PCORI Engagement Rubric to prioritize patient centeredness and facilitate actionable feedback from participants during the RCT planning phase (35). FG sessions included a short slide presentation in which study team members with training and experience in facilitation of focus groups (A.K. and L.B.) presented various aspects of the intervention and recruitment/retention plans. Topics presented during each FG are detailed below. Feedback was elicited from the HEALERS throughout and at the conclusion of each FG via open-ended questions. VW specialists were available for questions specific to the VW platform. FGs were audio and video-recorded and field notes were made by the facilitators during the FGs. See Figure 3 for an overview of the FGs. The Consolidated Criteria for Reporting Qualitative Research (COREQ) checklist was utilized to ensure comprehensive reporting of the methodology and findings (37).

The HEALERS were provided with access to the *Destination Cardiac Rehab* platform within Second Life® and created individual avatars. They explored the VW platform individually and also met as a group on the platform during session 5 (detailed below).

#### Focus group session 1

The initial session oriented members to the study team and the VWCR intervention. The study team and the HEALERS participants introduced themselves and described their motivations for the research project and joining the advisory board respectively. Based on the PCORI engagement principles “reciprocal relationships” and “partnerships”, the group set ground rules for the meetings, proposed a meeting schedule, and discussed general expectations and goals. A commitment was made to open and honest communication. The study team described *Destination Cardiac Rehab* and the VW platform in detail.

#### Focus group session 2

The HEALERS were presented with an overview of the disparities in CR participation and aims of the VWCR RCT. The components of the VWCR intervention were reviewed again in detail including the planned education session topics. Lastly, the group was shown 20 *Destination Cardiac Rehab* logo designs and voted via an online poll to determine the final logo (previously published 32) which will be included on all study materials.

#### Focus group session 3

Members were provided with a glimpse into the patient experience of an NC visit. An NC presented the components of the NC visits including a detailed review of the ITP and physical and mental/emotional health screenings. One of the study team members (A.K.) and the NC performed a mock NC visit through role play. Additionally, the plan for recruiting participants was reviewed with the HEALERS. The group watched two CBCR program video recruitment examples from partnering sites and collaborators. The HEALERS provided feedback regarding the NC visits, recruitment strategy overview, and recruitment videos.

#### Focus group session 4

Guest EPs detailed the components of an EP visit including development/refinement of a home-based exercise prescription and review of the ITP, adherence to the exercise plan, new/concerning symptoms, and concepts from the VWCR curriculum. The two EPs demonstrated a typical EP visit. The study team also presented the retention strategy which includes assembling a diverse study staff, offering high-quality services such as convenient assessment times/places, sending study activity reminders via preferred contact method, acknowledging and appreciating participants, and providing motivational rewards and incentives as detailed in Figure 2.

#### Focus group session 5

The PCS-AB members provided final feedback regarding the trial plan via videoconferencing. The group then moved to the *Destination Cardiac Rehab* platform where the members provided real-time feedback on the VWCR features while navigating throughout the platform with the study team.

### Data analysis

The study team reviewed the recordings and the participant feedback portions of the recordings were transcribed for data analysis. Participant identifiers were removed and replaced with a random number. Two study team members (H.A. and G.A.) independently read the transcripts multiple times for familiarization. A coding framework with predefined codes based on the research objectives – recruitment, intervention, and retention strategies – guided the initial analysis. Following this, an inductive approach, which involves gathering participants' insights and experiences, without predefined categories to allow themes to emerge naturally from discussion, was used to facilitate an open discussion probing participants' experiences (38, 39). The agenda evolved as insights arose from the discussion. Themes and subthemes were analyzed within each FG and compared across sessions to identify recurring patterns by content analysis (38–40). Illustrative quotes were selected to contextualize the major themes identified. Discrepancies were resolved by discussion with the study principal investigator (L.B.) until consensus was reached. Key themes and insights were analyzed to identify actionable insights to inform further refinement of the trial protocol.

## Results

### Participants

The HEALERS advisory board included 22 members with representatives from all six study-sites. There was 100% retention rate with no members dropping out. Members had a mean age of 59.3 ± 13 years (range 28–77 years) and 50% self-identified as women. The group includes members from diverse racial and ethnic backgrounds with 59% of participants self-identifying as White, 23% Black or African American, 9% Asian Indian, 4% Asian Filipino, 9% Hispanic or Latino, and 4% did not report. Randomly assigned participant numbers and associated demographic information are listed in Table 1. The majority of members were patients who previously completed a CBCR program for a variety of indications including acute coronary syndrome (e.g., myocardial infarction) [*n* = 5], coronary artery bypass surgery [*n* = 1], heart failure [*n* = 1], heart transplant [*n* = 3], and percutaneous coronary intervention [*n* = 1]). Seven members were patients who did not disclose their CR indication. The remaining members in addition to some of the patients described diverse work and personal experiences including work in healthcare through direct patient care, CR administration, clinical research, information technology, hospital volunteering, and support organizations for patients with heart disease.

**Table 1:** Deidentified participant numbers and corresponding demographic information.

Participant number	Age	Sex	Race	Ethnicity
1	66	Male	White	Hispanic or Latino
2	70	Female	Black or African American	Not Hispanic or Latino
3	56	Female	White	Not Hispanic or Latino
4	70	Male	White	Not Hispanic or Latino
5	53	Female	White	Not Hispanic or Latino
6	76	Male	White	Not Hispanic or Latino
7	53	Male	White	Not Hispanic or Latino
8	61	Male	White	Not Hispanic or Latino
9	67	Male	White	Not Hispanic or Latino
10	45	Male	Unknown	Hispanic or Latino (Mexican)
11	60	Female	White	Not Hispanic or Latino
12	28	Female	Asian Filipino	Not Hispanic or Latino
13	76	Male	White	Not Hispanic or Latino
14	61	Female	White	Not Hispanic or Latino
15	69	Male	White	Not Hispanic or Latino
16	53	Female	Black or African American	Not Hispanic or Latino
17	77	Female	Black or African American	Not Hispanic or Latino
18	38	Female	White	Not Hispanic or Latino
19	42	Male	Asian Indian	Not Hispanic or Latino
20	58	Male	Asian Indian	Not Hispanic or Latino
21	54	Female	Black or African American	Not Hispanic or Latino
22	73	Female	Black or African American	Not Hispanic or Latino

### Motivations for joining the HEALERS

Members cited a variety of motivations for joining the HEALERS. Several members expressed a desire to help those undergoing similar experiences to their own and paving a smoother path for others. Several participants experienced a long waiting period for CR availability and hoped programs like *Destination Cardiac Rehab* can provide more expedient care. Others felt a need to give back to the healthcare community in appreciation for the care received after their cardiac event. Many members joined due to a personal curiosity and interest in the intervention. Lastly, participants who work in CR administration and work with support group organizations joined to share their unique perspectives simply to assist in the development of an alternative CR program. Illustrative quotes are listed by participant in Table 2.

**Table 2:** Motivations for joining the HEALERS.

Participant	Illustrative quote
3	Participant 3 recently received a heart transplant and noted, “*In my outside work, I work in clinical research. So, I have a passion for clinical research.*”
10	Participant 10 who works in Information Technology stated, “*I'm just curious about how the VW would be used in a CR setting and what all that is. So, I just wanted to find out what it is and see if I could help.*”
12	Participant 12 who works as a bedside nurse stated, “*…I want to be able to learn about it so I can possibly suggest it to patients I come across… Having done CR myself, I found it very helpful. It helped me improve my condition a lot and helped me get back to work. So, I think it will be helpful a lot to other people.*”
14	Participant 14 waited 8-months to start CR stated, “*A strong reason to support a program like this is so that more people can get going sooner.*”
15	Participant 15 stated, “*I would have sign-up for this sort of thing had it been available. I waited over two months to get into their CR program here. So I'm all for anything you can do by yourself.*”

CR, cardiac rehabilitation; VW, virtual world.

### Destination Cardiac Rehab logo

**Figure 4:**
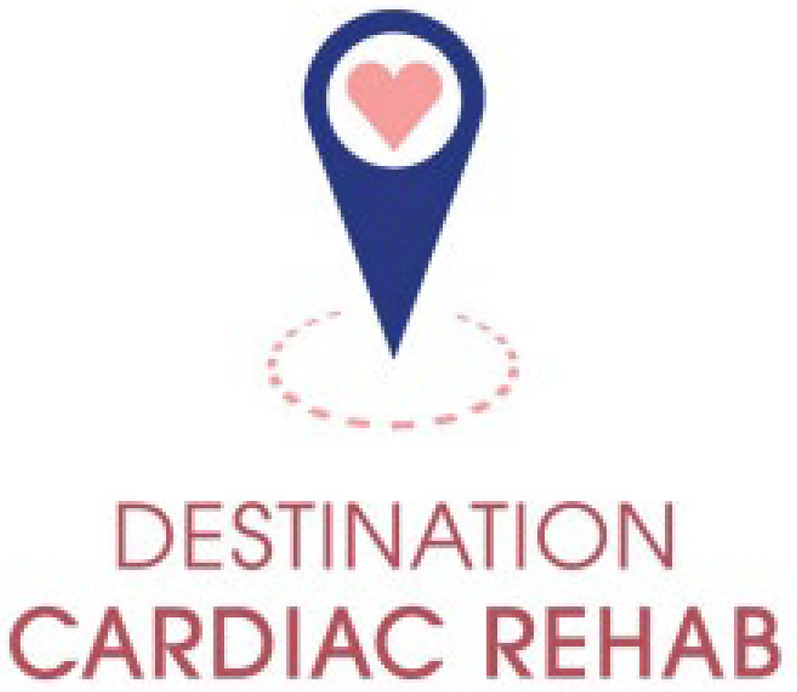
*Destination Cardiac Rehab* logo selected by the HEALERS. Created with adobe illustrator.

The HEALERS voted on a logo via online poll to represent *Destination Cardiac Rehab.* The logo depicted in Figure 4 was chosen by popular vote.

### Findings

Major themes were identified and categorized into groups: recruitment strategy, VWCR intervention and platform, and retention strategy. Themes and subthemes are summarized in Table 3 and illustrative quotes for each subtheme in Tables 4–6.

**Table 3:** Summary of themes and subthemes from focus group discussions.

Group	Theme	Subtheme
Recruitment strategy	Communication strategies	CR as part of the treatment plan
		CR is for everyone
	Recruitment video	Include VW footage
		Patient testimonials
		Short and concise
	Recruitment setting	Recruit from support groups
		Alert outpatient cardiologists of the program to discuss with patients
VWCR intervention	Safety	Exercise monitoring
		Monitoring devices
	Social support	Importance of social support
		Real-life meetups of VWCR participants
	NC and EP visits	Adequate visit time
		Focus on nutrition
		Integrated diet log
	VWCR platform	Platform availability on tablets and smartphones
Retention strategy	Reminders	Reminder messages per patient preferences (e.g., email, patient portal, etc.)
	Incentives	Provide tangible real-world and VW incentives for completing milestones
	Relationships	Relationships with study team members and other patients

CR, cardiac rehabilitation; EP, exercise physiologist; NC, nurse coach; VW, virtual-world.

**Table 4:** Illustrative quotes of member recommendations to optimize the recruitment strategy.

Theme	Subtheme	Illustrative quotes
Communication strategies	CR as part of the treatment plan	*1. “I had my [cardiac event] … and they said okay, you have to start CR. I didn't think it was an option. I just thought I had to do this if I wanted to get better.”* The same participant subsequently offered, “*…it should maybe be put a different way; just like [with a] shoulder injury or spine fusion, you spend 4 months in physical therapy. It's almost mandatory. You cannot be without it and fully recover… it's just not an option.”* (Participant 8, FG 2) *2. “I know when I was in CR, there was someone there in a wheelchair. Even though they weren't as mobile as other folks, they would still work on the machine and they would see improvement. How do we get people to understand that no matter how mobile you are, this is still something you should do?”* (Participant 10, FG 2)
Recruitment video	Patient testimonials/Including VW footage	*1. “And it is… it's for everyone that has had some type of cardiac event. So, they're not specifically saying that it's for people that just had a heart attack or somebody that just had some type of uh… well they're just not excluding anyone basically.”* (Participant 2, FG 3)
	Short and concise	*1. “Some people don't want to hear a long spiel of information. They just want something short and sweet to say what is CR… some people are only gonna listen to short and sweet.”* (Participant 10, FG 3)
Recruitment setting	Recruit from support groups	*1. “I am part of a couple of online support groups for [my cardiac condition] that has 3,000–3,500 people in it and they are suffering so much…. It would be really easy to reach out to those groups as well as far as doing intake for recruiting for the clinical trials. And I think it would be nice to have some people with my condition represented here.”* (Participant 14, FG 3)
	Alert outpatient cardiologists of the program to discuss with patients	*1. “I wasn't offered CR until later after I saw my cardiologist for the first time. I know you said the study coordinators will be there either before they check out of the hospital… but I think the cardiologist would be great to offer the patients the option for the trial. Because, well there wasn't one going on at the time so I don't know if she would have had she known about a clinical trial like this* vs. *just do you want to go to CR.”* (Participant 11, FG 4)

CR, cardiac rehabilitation; FG, focus group.

**Table 5:** Illustrative quotes of member recommendations to optimize the VWCR intervention.

Theme	Subtheme	Illustrative quotes
Safety	Exercise monitoring	*1. “I think the thing that kept me going was you know, once you get there, they hook you up to the EKG and they're monitoring you the whole time when you're exercising… It was kind of a fear-based thing that I got over after 36 sessions; just knowing that you're being monitored and that it's okay for your heart rate to get up, to be breathing heavy, and sweat a little bit…”* (Participant 11, FG 2) *2. “I think that the confidence you build by being attached to equipment and [EPs] are with you while you're exercising and can monitor you… to me that's huge, that's one of the most important reasons I felt good about going.”* (Participant 11, FG 4) *3. “When I start doing the exercises, I know that the questions come up… well I start to feel like my chest starts to hurt or other little symptoms… So, from my experience… you're on a treadmill or stationary bike and they're actively monitoring your HR and taking your BP after so many minutes of activity. Would those types of things be done? Or how are they being done with folks that are doing this virtually?”* (Participant 10, FG 4)
	Monitoring devices	*1. “I'm finding that for me in my condition, I struggle with weight management. And it's not just the weight from eating, it's the water weight as well. And I'll just give you an idea, between yesterday and today there was a 5-pound swing. Which of course has to do with water retention… So, I weigh myself and I find that managing the weight is crucial… So, part of the tools I think should be an accurate scale.”* (Participant 8, FG 2)
Social support	Importance of social support	*1. “If there's a group, I can easily connect with them. When I was going to inpatient rehab, I couldn't wait to get there because there were people there with the same goals or similar goals that I had that was just the social aspect of exercising and we all the same goal of getting healthier. But when you're doing it by yourself, it's kind of hard.”* (Participant 17, FG 4)
	Real-life meetups of VWCR participants	*1. “I think finding a workout buddy or partner is very important because I know he's going to be waiting for me in the parking lot at 7:30 and vice versa. So, I think having somebody to workout with makes a big difference. Just having someone to motivate you.”* (Participant 1, FG 2)
NC and EP visits	Adequate visit time	*1. “The length of that conversation between the two of you was probably as short as one can be with a cardiac patient. Because when we have someone's ear, we tend to really want to take advantage of that. We don't want to go around talking about our heart issues with everybody. But when someone asks, it's our opportunity to talk. So, allotting a little more time is probably not a bad idea.”* (Participant 14, FG 3)
	Focus on nutrition	*1. “The nurses ask, “how is your salt intake?” and the patients say, “oh it's pretty good” instead of saying this, [they should be saying] how many milligrams of sodium per day you should be having. So, your BP is 180/100 mmHg and they probably need a little more direction about how much sodium.”* (Participant 14, FG 3) *2. “It would be great to have concrete examples for diet”.* (Participant 16, FG 3) *3. “I didn't realize how noncompliant I was with a heart healthy diet. I think I'm going to start logging my actual food intake and that'll give me more insight and more motivation.”* (Participant 2, FG 3)
	Integrated diet log	*1. “ Inside the app for the Fitbit, there is the diet diary portion in there. So definitely using the Fitbit and using the tools that are in there, that would be definitely something to do.”* (Participant 10, FG 3)
VWCR platform	Platform availability on tablets and smartphones	*1. “It's not always practical to travel with a laptop and I travel a lot. I very often take my big iPad and just fold it and I can work… I can work on everything. With that platform, as opposed to other things, I would have to take my [laptop]. What is the future for it to be on an iOS platform for example?”* (Participant 8, FG 5)
	Positive feedback	*1. “…When I was in [the VWCR] a few days ago and I went into the restaurant and I was doing the whole menu order thing…it came up with a text box in the corner that said well this is what you would have eaten calorie-wise and this is what it takes to burn that off in the gym and it was huge numbers. I was really shocked… But to get that feedback on you know, I ordered way too much at the Chinese restaurant and I should have probably ordered only a quarter of what I ended up… That to me is what is going to be very empowering and very helpful going forward in trying to avoid being back in the hospital again later on.”* (Participant 11, FG 5) *2. “Using the gym was exciting because it was virtual reality… like you said, that's what it is. And I had a chance to do exercises that I don't think I could do otherwise. Because my joints are not that limber and I'm not that strong. But it made me feel like I could do it. Especially like the Pilates. I think I did an advanced way of doing the Pilates, and that was exciting. I would love to be able to get into Pilates more than just stretching movements. But that was exciting to do.”* (Participant 2, FG 5)

CR, cardiac rehabilitation; EP, exercise physiologist; FG, focus group; VWCR, virtual world-based cardiac rehabilitation.

**Table 6:** Illustrative quotes of member recommendations to optimize the retention strategy.

Theme	Subtheme	Illustrative quotes
*Incentives*	Provide tangible real-world and VW incentives to completing milestones	1. “*…I really think there is something about getting some kind of reward at certain points along the way… So, I do like that you're considering a little bit of some kind of motivation for reaching pivotal points in the process. Something to keep working for… even if it's just bragging rights…”* (Participant 14, FG 4) *2. “Maybe if there were some types of incentives [within the VW], like a little cap or a certain type of badges. Like something on your avatar that could say something like… I did a certain type of exercise this week or I've been through half the program or that kind of thing. A virtual incentive.”* (Participant 10, FG 4)
*Relationships*	Relationships with study team members and other patients	*1. “I mentioned that I was going to go for 7–8 [CR sessions] and then I stayed for 36. I told the staff this, the EPs, that's the reason I stayed was for them. They were so dedicated to what they were doing. You know, the way they cared for me and for the other people in the room, I would have felt bad walking away from the effort that they put in.”* (Participant 6, FG 2) *2. “What would make me more interested than any of this stuff would be contact/interaction with the people, like the EPs and the feedback directly to your cardiologist if needed. So those types of things made it much more important that I attend the CR classes because I get so much more feedback on that sort of stuff.”* (Participant 11, FG 2)

CR, cardiac rehabilitation; EP, exercise physiologist; FG, focus group.

#### Recruitment strategy

##### Communication strategies

Participants discussed the impact of the communication strategies utilized in their CBCR recruitment experiences and how those strategies impacted their decisions to enroll. Participants noted that their care teams conveyed CR as a mandatory component of their care plan which motivated them to participate. They recommended conveying that CR is necessary regardless of baseline physical functioning. Based on these observations, the group agreed that the study overview and recruitment videos should (1) endorse CR as an essential part of the treatment plan and (2) emphasize its use in all patients regardless of physical functioning.

##### Recruitment video

Based on the two recruitment videos shown, participants preferred the shorter video. Participant 10 suggested using footage from the VWCR platform and including prior patient testimonials. Participant 10 also pointed out that one of the videos stated that CR is for everyone with cardiac diseases and not just those who had heart attacks and emphasized that the recruitment video should highlight this.

##### Recruitment setting

In addition to recruiting patients during their index hospitalization and from the outpatient CR census list, participants recommended additional recruitment settings. For example, Participant 14 recommended recruitment from support groups for patients with rare cardiac conditions. Additionally, Participant 11 recommended alerting outpatient cardiologists of the trial to expand recruitment.

#### VWCR intervention

##### Safety

Several participants across multiple FGs expressed low confidence in exercise capacity and exercise safety after their cardiac event and the need to be monitored during exercise to regain that confidence. The participants stressed the need for a similar mechanism for real-time symptom reporting to ensure exercise safety and rebuild confidence. In addition to the PA tracker and blood pressure (BP) monitor, Participant 8 suggested including an accurate scale so that participants can report daily weights to the study team to alert the team of early decompensated heart failure.

##### Social support

In addition to ensuring adequate real-time exercise monitoring, participants emphasized the importance of the social support garnered in CBCR. Participant 17 recommended that the study team compile a list of community resources that offer group exercise to simulate this experience. Other participants echoed this suggestion. Additionally, Participants 1 and 9 shared that they met during CBCR and continue to exercise together 3-times per week. Participant 1 highlighted the importance of this relationship for maintaining accountability*.* Based on this feedback, the participants suggested providing a mechanism for patients in the VWCR group to share their contact information to allow those in close proximity to establish similar relationships.

##### NC and EP visits

Participants emphasized the importance of allotting adequate visit time to ensure the patients are being heard. Additionally, multiple participants expressed that their CR programs provided vague nutrition recommendations and suggested that one of the NC visits focus on nutrition with clearer guidance. Participant 10 pointed out that there is a built-in diet log within the PA tracker that may be useful to review during NC visits. Other participants reported that their CBCR programs included a nutritionist and/or provided links to a video that provided specific nutrition recommendations, which they suggested may be viable alternatives to including detailed nutrition advice during NC visits.

##### VWCR platform

The advisory board members gave very positive feedback regarding the VWCR platform and had few recommendations for revision. Participant 8 asked whether the VWCR platform could be made available on tablets/smartphones.

#### Retention strategy

##### Reminders and incentives

Several participants endorsed receiving text messages and portal messages during CBCR, which they found helpful. They did not have any additional suggestions to add to the current plan. They did stress the importance of incentive materials in motivating participants. Participant 14 suggested commemorative rewards (such as T-shirts, coffee mugs, etc.) when reaching milestones. Participant 10 suggested including rewards within the VWCR platform such as caps or badges the avatars could wear.

##### Relationships

Multiple participants noted that the relationships formed during CR was the most important factor that motivated them to persevere. They mentioned a sense of accountability to the CR staff in addition to their peers in the CR program. This accountability not only motivated them to complete all 36 CR sessions but also continue healthy lifestyle changes beyond the CR program.

## Discussion

Utilizing CER methods, we successfully recruited a diverse PCS-AB and conducted five FGs to inform iterative refinements to *Destination Cardiac Rehab* and the recruitment/retention strategies for an upcoming RCT which will compare adherence and efficacy of this novel intervention to traditional CBCR (41, 42). The HEALERS offered important actionable insights, recommendations, and concerns that the study team analyzed to inform changes to *Destination Cardiac Rehab* and the trial protocol to better meet patients' needs. In addition to a growing body of literature regarding CER, our findings highlight the immense value in including patients, community members, and key stakeholders in the design process of novel interventions to better understand what is important to patients and how to best meet their needs (2). Refinements to the *Destination Cardiac Rehab* RCT protocol made based on the HEALER's feedback are detailed in Table 7.

**Table 7:** Refinements to Destination Cardiac Rehab based on the HEALERS feedback.

Feedback	Refinements
Using clear language	• Recruitment video was edited with clearer language
Broadening recruitment settings	• The study team will utilize support groups and alert outpatient cardiologists of the program to broaden recruitment
Supplying a scale	• Participants in the trial will be provided a scale to measure their weight and share with the study team
Incorporating explicit nutritional counseling	• Education sessions regarding nutritional counseling were revised based on the HEALER's feedback
Sending reminder messages	• The study team will send reminder messages via participants' preferred communication method for upcoming sessions
Providing tangible incentives throughout the program	• Participants will be provided with resistance bands, BP monitors, and Fitbits • Gift cards will be provided throughout the program
Perceived safety concerns in performing exercise at home with direct in-person monitoring	• Multiple touch points with study team members including NCs and EPs • Participants will be provided with PA trackers and BP monitors and measurements will be provided to team members • Triage system created to direct follow up care if new symptoms arise
Limited social support with VWCR compared to CBCR	• Weekly virtual support group will be available to provide ongoing social support within the VW • Participants will be provided with a list of fitness centers/gyms with group exercise sessions in each study site location • Participants may voluntarily share contact information with others in the group to support ongoing relationships beyond the 12-weeks of structured CR

BP, blood pressure; CR, cardiac rehabilitation; EP, exercise physiologist; NC, nurse coach; PA, physical activity; VW, virtual world.

Many recommendations by the HEALERS can be readily implemented into the existing protocol including: using clear language, broadening recruitment settings, supplying a scale, incorporating explicit nutritional counseling, sending reminder messages, and providing tangible incentives throughout the program. Two major concerns raised throughout the sessions were (1) perceived safety concerns in performing exercise at home without direct in-person monitoring and (2) limited social support with VWCR compared to CBCR. These issues require multifaceted solutions, as discussed below, to best simulate the positive aspects of the CBCR experiences described by the HEALERS that are inherently absent in VWCR and mitigate any impact their absence may have on patient motivation/adherence.

HEALERS members that previously participated in CBCR reported that in-person exercise monitoring in their CBCR programs offered a sense of security that bolstered their confidence and motivated them to persevere. Despite sufficient literature to support the safety of alternative CR programs without direct exercise supervision in low- to moderate-risk patients, they worry that the absence of that perceived security may hinder progression of exercise goals (23, 43). While our prior proof-of-concept and pilot studies demonstrated excellent adherence, participants simultaneously participated in CBCR. Thus, the safety concerns highlighted by the HEALERS were not present in our prior studies (33). However, prior studies directly comparing HBCR and CBCR suggest that the absence of direct supervision during exercise in HBCR does not negatively impact participation rates or improvement in functional capacity (23, 44). According to Velez et al. within a systematic review of qualitative analyses of telerehabilitation programs, patients that participated in telerehabilitation programs reported a sense of empowerment to take ownership of their own rehabilitation journey which facilitated achievement of their health and lifestyle goals (45, 46). Nevertheless, we acknowledge the concern raised by the HEALERS and have included multiple mechanisms to alleviate potential feelings of unease. Although the nature of our intervention precludes direct supervision of exercise, our intervention includes multiple touch points with study staff in which patients can share new symptoms or other concerns they may have during exercise. Additionally, patients will be provided with PA trackers which will monitor heart rate during exercise. Patients will also be equipped with BP monitors and encouraged to take BP measurements prior to and following independent exercise for review with the EP and NC at weekly visits. The EPs will discuss in detail normal symptoms during exercise and symptoms that should raise an alarm and compel the patient to discontinue exercise and/or present to urgent medical attention. Lastly, there is a triage system in place to direct follow up-care if new symptoms arise. Based on prior studies and these planned safeguards, we expect that patients will persevere in the absence of direct exercise supervision.

The HEALERS also mentioned on several occasions that the social support/network and relationships they built during CBCR motivated them to maintain behavioral change during their structured CR programs and beyond. Several participants continue to exercise regularly with people they met in CR and expressed concern that this may not be possible for patients who participate in *Destination Cardiac Rehab*. This sentiment is supported by multiple studies which show that the absence of in-person social interactions negatively impacted patients' rehabilitation experiences (45, 47, 48). *Destination Cardiac Rehab* has many unique features designed to address the highly important social support aspect of CR that is often missing in HBCR programs. Most notably, *Destination Cardiac Rehab* capitalizes on simulating in-person experiences via a virtual platform. Additionally, the weekly virtual peer support group meetings which were specifically designed to foster relationships among patients have been shown in our prior studies to be very effective in garnering social support (32, 33, 49). The patients will also have very frequent touch points with the VWCR staff via videoconferencing (equivalent to those participating in CBCR) which we expect to cultivate similar relationships with CR staff as those described by members of the HEALERS. In response to feedback by the advisory board, we will compile a list of fitness centers/gyms with group exercise sessions in each study site location for those who seek in-person group exercise experiences. We will also offer a mechanism for patients to voluntarily share contact information with others in the group to support ongoing relationships beyond the 12-weeks of structured CR.

Overall, the group shared their personal experiences, viewpoints, and opinions to ensure that patients with similar perspectives who will ultimately benefit from *Destination Cardiac Rehab* are represented in its design and implementation. The HEALERS will continue to meet throughout the RCT to inform implementation and dissemination strategies.

## Limitations

Our study has several limitations. First, the PCS-AB members are relatively young (average age 59 years-old) and older individuals with more barriers to technological interventions may be underrepresented (50). An inherent limitation in focus groups is potential imbalances among the group in assertiveness and openness to share their experiences, some participants' perspectives may be disproportionately represented. While some participants were less assertive or open, all of the members participated in the discussion. Lastly, the study team was unable to recruit participants who have been candidates for CR but did not participate. Most of the members who had previously participated in CBCR participated in the majority of CBCR sessions and may not represent individuals with greater barriers to CR participation. Nevertheless, they offered valuable insight into their own CR experiences and the aspects of their programs that fostered motivation and perseverance.

## Conclusion

The HEALERS, a community advisory board comprised of patients, community members, and key stakeholders was successfully convened and FG sessions were held to collaborate with the *Destination Cardiac Rehab* study team to offer insights to inform improvements to the intervention and implementation plans in anticipation of a patient and community-centric RCT. The HEALERS recommended using clear language, broadening recruitment settings, supplying a weight scale, incorporating explicit nutritional counseling, sending reminder messages, and providing tangible incentives throughout the program. They also noted two very important concerns: (1) perceived safety concerns in performing exercise at home without direct in-person monitoring and (2) limited social support with VWCR compared to CBCR and potential solutions to address these concerns. Our study highlights the value of CER approaches in garnering community member feedback to ensure representation of diverse perspectives in the design and implementation of a novel intervention.

## Data Availability

The raw data supporting the conclusions of this article will be made available by the authors, without undue reservation.
